# Type III B endoleak leading to aortic rupture after endovascular repair: analysis of errors in follow up and treatment

**DOI:** 10.1186/s42155-018-0020-6

**Published:** 2018-11-06

**Authors:** Marco Leopardi, Alessia Salerno, Pietro Scarpelli, Marco Ventura

**Affiliations:** Department of Vascular Surgery Unit, San Salvatore Hospital – University of L’Aquila, Via L. Natali, 67100 L’Aquila, Italy

**Keywords:** Type III B endoleak, Aortic rupture, Fabric rupture

## Abstract

**Background:**

The aim of this paper is to describe the case of a patient with a type III endoleak which was misdiagnosed and treated without success as a type I-II endoleak. An incorrect endoleak diagnosis lead to aortic rupture, which could be avoided with a correct diagnosis. Type III B endoleaks presents some diagnostic difficulties, in the case we describe, they were increased by late presentation and poor follow up.

**Case presentation:**

We revised this 89 years old patient history, he underwent EVAR 11 years before, a control scan six month after surgery, showed a type I-II endoleak which was still present after first intervention. He was treated with proximal cuff positioning and embolization coils. Eight years after first intervention, a Computed Tomography Angiography (CTA) showed persisting type I-II endoleak so same problem was suspected and patient was treated with another proximal cuff and right iliac extension. A Magnetic Resonance Imaging (MRI) control, six months later, showed an increase of the aneurysm sac size of 12 mm. Two years later patient presented at emergency room at our hospital with malaise, sweating and abdominal pain. Computed Tomography (CT-scan) showed increased abdominal aortic diameter (140 × 130 mm) with rupture and hemoperitoneum. He was treated in urgent fashion with endograft removal and aortic-iliac Dacron graft reconstruction. During surgery three large tears on endograft fabric and a stent suture rupture were observed. After surgery patient was admitted in intensive care unit and died on second postoperative day due to multiorgan failure.

**Conclusions:**

Type III endoleak is an uncommon complication: a correct and prompt diagnosis is mandatory for appropriate treatment After EVAR, and especially in those cases of known endoleak, a correct follow-up is mandatory and in case of diagnostic doubts correct imaging should be performed. Media contrast allergies should not be neglected and should not represent a CTA limitation.

## Background

Type III Endoleak is an important and quite rare complication after Endovascular Aortic Repair (EVAR), as its incidence range is reported around 3–4,5% (Lal et al., 2015).

As Type III A is defined as graft components disconnection and consequent leakage, Type III B is described as a graft fabric defect and it presents some diagnostic challenges, and only its precise diagnosis allows an effective treatment.

The aim of this paper is to describe the case of a patient with a type III endoleak which was misdiagnosed and treated without success as a type I-II endoleak. An incorrect endoleak diagnosis lead to aortic rupture, which could be avoided with a correct diagnosis. Type III B endoleaks presents some diagnostic difficulties, in the case we describe, they were increased by late presentation and poor follow up.

We describe the case of a patient with acute aortic rupture 11 years after EVAR, with a diagnosed endotension, which was judged untreatable due its age and comorbidities. So he underwent emergent surgical conversion with aorto-iliac reconstruction.

## Case presentation

An 89-years old patient presented at Emergency Room at our Hospital with malaise, sweating and abdominal pain. He previously had EVAR 11 years before in another institution, which was poorly controlled at follow up due a media-contrast allergy.

He was implanted Talent Medtronic graft (Medtronic Minneapolis, MN, USA) with bilateral surgical femoral access, in another hospital, for that reason we have very few informations from patient relatives and patient discharge report.

A control Computed Tomography Angiography (CTA) 6 month after surgery, showed a type I-II endoleak, which was still present after first intervention, that was promptly treated in the same institution with proximal cuff positioning and embolization coils. Patient is then lost at follow-up for 8 years, when he had another CTA that showed persisting type I-II endoleak. He was treated with another proximal cuff and right iliac extension.

Patient had consequently a magnetic resonance imaging (MRI) control 6 months later, showed an increase of the aneurysm sac size of 12 mm, at that time referring physician preferred MRI for media-contrast allergy. At that point referring vascular surgeon in the prior institution diagnosed that condition with endotension, with the unique possibility to treat the patient with an open conversion, as he had already several inconclusive endovascular treatments. But patient age of 87 years, and other comorbidities contraindicated open conversion, limiting surgeons to patient observation. Patient never had a new diagnostic angiography.

**Fig. 1:**
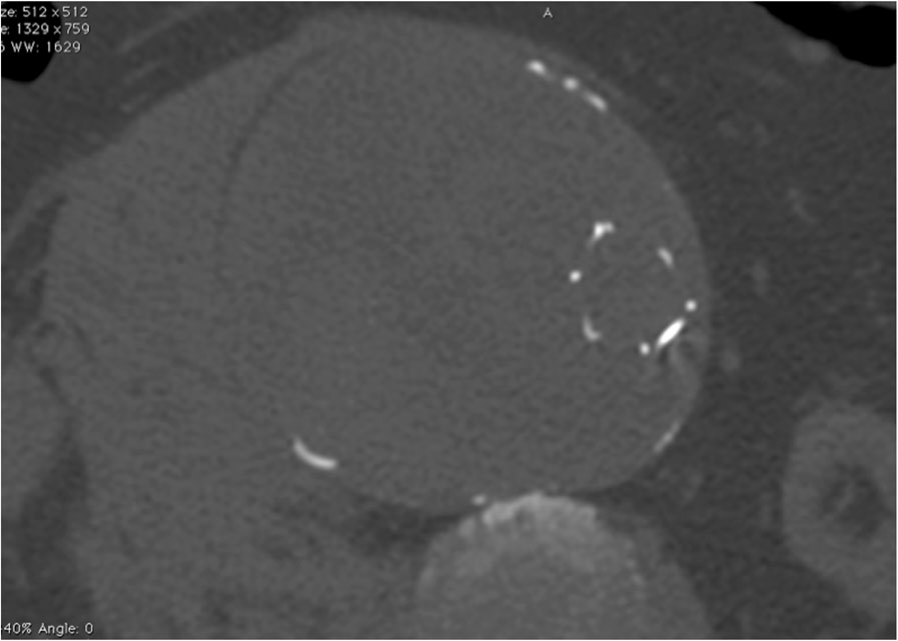
CT-scan without media-contrast, at Emergency room which showed a large increase of abdominal aortic diameter (140 × 130 mm) with rupture and hemoperitoneum. CT-scan: Computed Tomography scan

Two years later, when he presented at Emergency Room at our hospital, conditions rapidly worsened, a CT-scan without media-contrast for media-contrast allergy, showed a large increase of abdominal aortic diameter (140 × 130 mm) with rupture and hemoperitoneum. Fig. 1.

**Fig. 2:**
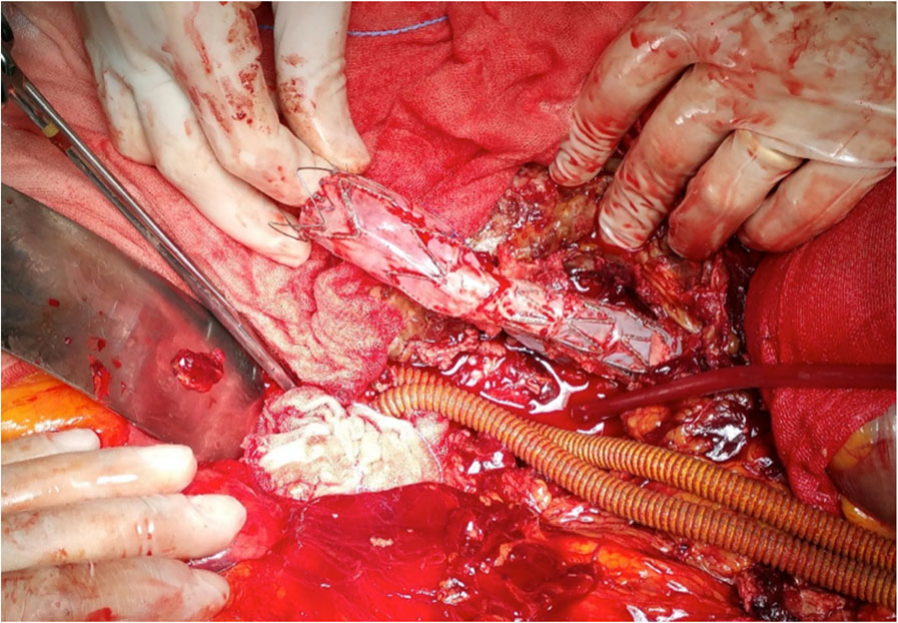
Proximal part of endovascular grafts removed, and bifurcated silver polyester graft after proximal anastomosis was realized

For that reasons, he was addressed to emergent surgery of EVAR conversion, with median laparotomy transperitoneal approach. An endovascular approach was not feasible, as in our institution we did not have a suitable endograft to promptly treat the patient. The endograft was removed but due to severe aortic pathology a supraceliac clamping was necessary. After all endovascular grafts were removed, proximal anastomosis between infrarenal aorta and bifurcated 16 × 8 mm InterGard silver polyester graft, (Maquet SARL, La Ciotat, France) was realized with 3/0 polypropylene suture. Fig. 2.

Distal anastomoses were completed on common iliac artery on the left and on common femoral artery on the right side, due iliac artery occlusion.

**Fig. 3:**
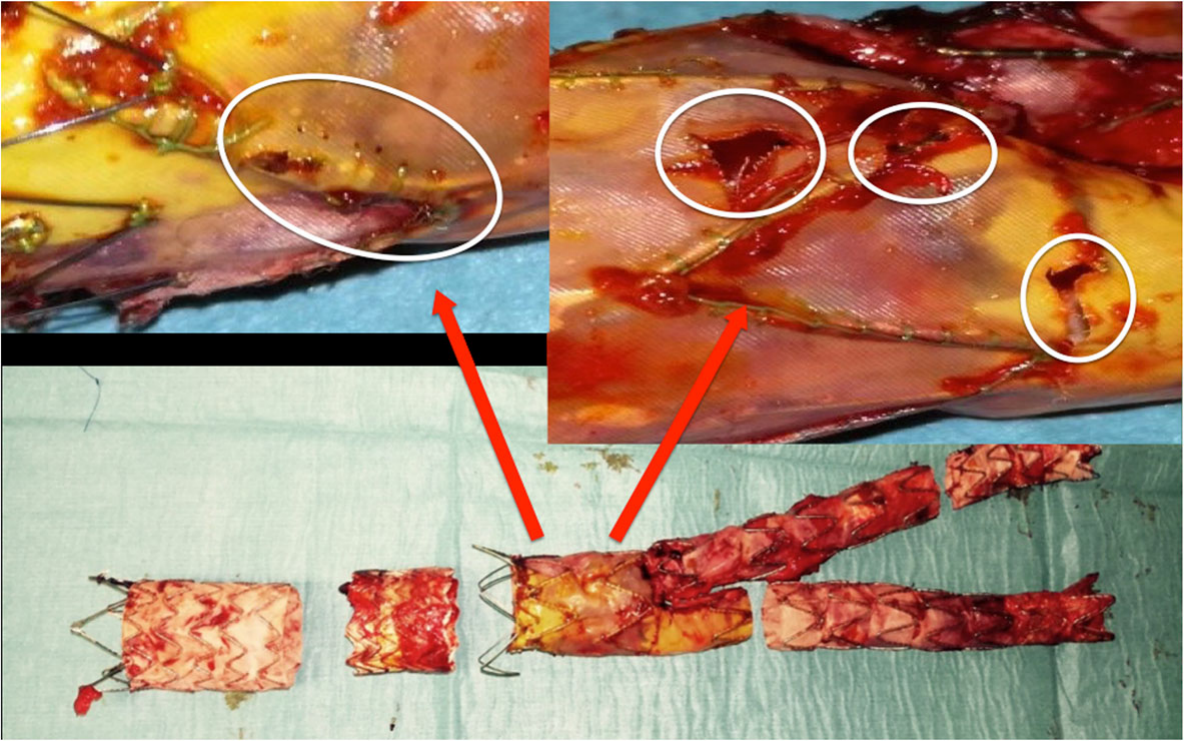
Endograft removed: tears on endograft fabric on the proximal portion of main body, the running suture of the first support stent was also broken

During surgery we observed three large tears on endograft fabric on the proximal portion of main body, each tear had irregular shape, a larger one with 3–4 mm diameter and other two smaller ruptures of 2 mm diameter. A running suture of the first support stent was also disrupted. Fig. 3.

During the whole intervention patient vital signs were stable, but critical, such as hypotension, tachycardia and anuria. Good flows were found at continuous wave ultrasound on distal arteries.

After surgery patient was admitted in intensive care unit, arterial tension was low nearly 50/20 mmHg, with absent diuresis. Arterial flow was present on distal arteries. Patient died on second postoperative day due to multiorgan fail.

## Discussion

The importance of a correct diagnosis is crucial for all endoleaks after EVAR and especially when dealing with type III endoleak, as a good treatment depends exclusively on diagnosis, as there are several surgical options with different invasiveness.

In our case the diagnosis seemed not correct as an endotension, defined as persisting blood in the aneurysmal sac, and was wrongly suspected due impossibility to find any source of leak at CTA. Iodine contrast allergy was also responsible of non-diagnostic scan, as contrast media was often avoided during follow-up.

Angiography was only performed in an early period of follow-up when he also had cuffs positioning and was not repeated when endoleaks was observed. Technical difficulties are mainly due to intrasac pressure, which avoids to clearly observing contrast media leak. CTA - is currently considered gold standard in endoleak diagnosis but angiography is the best exam to discriminate between endoleak types (Maleux et al., 2017).

According to our opinion in those cases with known endoleak, observing a yearly follow-up is mandatory, and in particular cases such endoleak in patients with iodine contrast allergy, which do not represent a limit of the exam and can be easily handled with pre-medication. When endoleak diagnosis brings to any doubts at CTA, or a type III endoleak is suspected, angiography must be performed: this allows to enhance imaging quality and gives information of intrasac flow.

Another method to diagnose endoleak is Doppler Ultrasound (DUS) and Contrast Enhanced Ultrasound (CEUS), they showed sensitivity of 72% and 91% and specificity of 95% and 89% for DUS for CEUS respectively, which suggest that those techniques may be used routinely, reserving CTA only after positive ultrasound. (Abraha et al., 2017) In our experience this method is currently used, but patient never had DUS examination during his follow up.

In case of media-contrast allergy alternative contrast agents, like carbon dioxide and gadolinium, have also been evaluated and can be used in specific cases. In particular carbon dioxide with its low viscosity seems to be effective for endoleak diagnosis and may be proposed in alternative to iodine contrast in case of allergies (Nadolski & Stavropoulos, 2013; Mascoli et al., 2018).

Type III B endoleak can be caused by several reasons, as seems difficult to assess the exact cause, some hypotheses can be considered: repeated endovascular manipulations, aortic ballooning, fabric damage by metal stent and manufacturing defects (Abouliatim et al., 2010).

Few cases are described in literature and are summarised in Table 1.

**Table 1:** Case reports found in literature

First Author	Years	N° of Patient	Age	AAA diameter (mm)	Type Of Endograft	Perioperative Complications	Signg Of Type III B Endoleak	Type III B Endoleak Etiology	Graft failure	Time From Evar (month)	Treatment Of Type III Endoleak
Wanhainen A.	2008	1	77	70	Zenith	No complications	Abdominal Pain	Unknown	Holes in the fabric on stent sutures	84	Relining with Exluder graft
Abouliatim I.	2010	1	81	60	Endurant	Type I Endoleak treated with re-ballooning	II post-operative day angiography	Repeated ballooning	Fabric rupture	0	Relining with Zenith endograft
Van der Vliet J. A.	2005	1	84	79	Zenith	Graft kinking treated with ballooning	Intraoperative angiography	Endovascular manipulation, excessive balloon pressure	Fabric rupture	0	Immediate relining
Brown E. K.	2008	1	89	57	Ancure	graft limbs kinking treated with Wallstents	DUS findins during surveillance	Repeated manipulations?	Unknown	72	Relining with aortouniliac Excluder graft
Teutelink, A	2003	2	78	54	Ancure	No complications	CT findins during surveillance	Unknown	Fabric rupture	36	Cuff relining with Excluder graft
			77	61	Ancure	Migration of the graft treated with Wallstent	CT findins during surveillance	Continuous strain by Wallstent	Fabric rupture	30	Open repair
Lee A. W.	2006	1	75	50	Excluder	Type IB EL treated with iliac extensions and reballooning	Pulsatility in abdomen	Repeated ballooning?	Unknown	12	Limb relining
Banno	2012	1	86	88	Zenith	Type IA EL treated with Palmaz stent	Lower back pain	Erosion from Palmaz stent?	Fabric rupture	27	Two proximal cuffs
Dayama A.	2013	1	71	67	Excluder	Type IA EL treated with Palmaz stent	Lower abdominal and back pain	Erosion from Palmaz stent?	Fabric rupture	60	Aortic Cuff and re-ballooning

A case of graft rupture is described after its ballooning, (Van der Vliet et al., 2005) in another case fabric erosion of an Ancure endograft (Guidant, Menlo Park, California) was caused by a “kissing” iliac Wallstents (Boston Scientific, Natick, Massachusetts) stenting placed after EVAR for limbs kinking, which caused erosion and over time. (Brown et al., 2008) Wanhainen described a case of ruptured aorta 7 years after EVAR (Zenith bifurcated endograft, Cook Inc., Bloomington, Ind) treated with open urgent repair; during surgery they observed several fabric tears on the main body, close to stent sutures (Wanhainen et al., 2008).

Teutelink reports a series of two cases of graft fabric rupture (both Ancure, Guidant), one with unknown cause and the other was referred to the fabric erosion caused by a Wallstent positioned during EVAR for a rotation-dependent stenosis of iliac limb. (Teutelink et al., 2003) Two cases describe multiple areas of fabric erosion, one under graft stent apices and one near multiple disconnection of nitinol frame with rupture of polypropylene sutures, both with same graft (Vanguard, Boston Scientific, Natick) (Beebe et al., 2001; Becquemin et al., 2002).

In other reported cases the cause of fabric tears was unknown and often described in the flow divider area, (Juszkat et al., 2009; Lee et al., 2006; Banno et al., 2012; Dayama et al., 2013) anyway there are no significant evidences in literature.

In our case we suspected that repeated endovascular manipulations might have gradually ruined endovascular graft with consequent leakage.

When a prompt diagnosis is achieved an endovascular, low-invasive treatment is deemed, such endovascular relining with Excluder bifurcated graft (W. L. Gore & Associates, Flagstaff, Ariz) to treat a type III endoleak after EVAR with Ancure graft (Guidant, Menlo Park, California) 3 years prior.(Beebe et al., 2001) Banno et al. report an aortic rupture 19 months after EVAR with Cook Zenith device, treated with a proximal cuff to cover fabric hole.(Dayama et al., 2013) Another complete relining was described with Gore Excluder graft but after open laparotomy direct graft observation, multiple minor bleedings were observed from holes in the fabric of the main body of a bifurcated Cook Zenith endograft, aorta was not clamped due patient’s general health status, with advanced age and Alzheimer disease (Teutelink et al., 2003).

Other cases describe a hybrid technique with aortomonoiliac endograft (Endurant II, Medtronic, or Zenith Renu, Cook) following a femoro-femoral crossover bypass.(Mascoli et al., 2018; Van der Vliet et al., 2005; Lee et al., 2006; Kansal & Nagpal, 2016) and one case reports a tentative of complete relining (Excluder, Gore) which was converted to aortouni-iliac configuration with a plug, due impossibility to cannulate contralateral gate.(Wanhainen et al., 2008) In another case where the type III B endoleak was coming from the proximal part of the ipsilateral iliac limb (Excluder, Gore) approximately 1 cm inferior to the top of the flow divider and it was treated with an iliac extension of the same type (Banno et al., 2012).

An open approach is obviously much more challenging in terms of mortality and morbidity, and technically more difficult as normally those patients may have been considered unfit for open surgery at the time of the initial EVAR.

Open repair is found in some series as urgent treatment after aortic rupture, (Becquemin et al., 2002) or as treatment of recurrent type III endoleak after a first endovascular attempt.(Mascoli et al., 2018) In another series open repair was indicated for patients with symptomatic endoleaks not amenable to endovascular repair or after unsuccessful endovascular attempts, (Perini et al., 2017) in one case no secondary endograft could be placed because of the size of the neck of the aorta, and open repair was necessary.(Beebe et al., 2001) Bequemin et al. report an open conversion due to the size of the aneurysm, the failure of numerous previous closure attempts, and the uncertain origin of the leak (Juszkat et al., 2009).

In our case an open conversion was necessary due the emergent condition of patient, we found relatively easy to approach endograft, also endograft removal was quite quick and simple, but this maneuver exposed the patient to an important blood loss, we found the more difficult part to deal with pathologic aorta and to finally obtain a good sealing anastomoses.

Moreover an endovascular emergent treatment requires a good *“*home” availability of several materials and a skilled team.

If type III endoleak was diagnosed prior rupture, an endovascular approach could have been realized, which could be more suitable for the poor general condition of patient and its age.

## Conclusions

Type III B endoleak is an uncommon complication: a correct and prompt diagnosis is mandatory for appropriate treatment.

After several years from first intervention and subsequent complications treatments it is impossible to determine the exact cause of graft ruptures but several repeated endovascular manipulations might have played a role. Poor follow-up conducted with scarce controls, and CT scan without media contrast did not help in discover exact type III B Endoleak etiology.

In those cases of known endoleak a correct follow-up is mandatory and contrast media allergies should not be neglected.

## Abbreviation

CEUS: Contrast Enhanced Ultrasound
CTA: Computed Tomography Angiography
CT-scan: Computed Tomography scan
DUS: Doppler Ultrasound
EVAR: Endovascular Aortic Repair
MRI: Magnetic Resonance Imaging
